# Supplementary material for: Effective coverage of antenatal care services in post war Tigray, Northern Ethiopia: An analysis of community and health facility–based surveys
Source: PLoS One. 2025 Oct 30;20(10):e0336121. doi: 10.1371/journal.pone.0336121 (PMC12574916; doi:10.1371/journal.pone.0336121)
Supplement: S1 File — (PDF) [file pone.0336121.s001.pdf]

Community based data collection tool (English version)

Information sheet and consent form

Information sheet

Hello! My name is _______________ I am a member of a data collector research conducting by Tigray Regional Health Bureau, Mekelle University College of Health Sciences, Tigray Health Research Institute (THRI), Adigrat University College of Health Sciences and Akusm University College of Health Sciences. The objective of this study is to improve and restore the health system in Tigray region including Maternal Health Care Services. I am one of the data collectors and I am going to ask you some questions related to Maternal Health Care Services. Your name will not be written in this form and will never be used in connection with any of the information you tell me. However, your honest answers to these questions will help to solving the problem on implementation of Maternal Health Care service in Tigray Region. The interview will take about 30 minutes and we are appreciating your help in responding to this research questions. You have a right not to participate and withdraw at any time of interview.

Would you be willing to participate? Yes……., No……

Consent form

I have been briefly informed about the study and clearly understood the objective of the study. So, I am agreeing to participate in this study.

Signature ………………………

Result

Result codes: Completed=1, Refused=2, partially completed=3, respondent no found=4

Socio-demographic information			
S.no	Question	**Response**	**Remark**
100	Zone: ________________<br>District: ______________<br>Tabia: _______________<br>Date of data collection: ________________		
101	Age of respondent (in completed years)	…………… (in years)	
102	Marital status of the respondent	1=Single<br>2= Married<br>3=Divorce<br>4=Widowed<br>5=Separated	
103	What is your educational status?	1=Unable to read and write<br>2=Able to read and write but not attend formal education<br>3=Elementary school<br>4=Secondary school<br>5= College and above	
104	What is your partner`s educational status?	1=Unable to read and write<br>2=Able to read and write but not attend formal education<br>3=Elementary school<br>4=Secondary school<br>5= College and above	
105	What is your religion?	1=Orthodox<br>2=Protestant<br>3=Muslim<br>4= Catholic<br>99=Others specify……….	

106	What is your ethnicity?	1= Tigrawayti<br>2= Erob<br>3= Afar<br>4=Amhara<br>99= Others specify………..	
107	What is your occupation?	1=House wife<br>2= Self-employed<br>3=Merchant<br>4=Governmental employed<br>5= Daily laborer<br>6= Farmer<br>99=Others specify…….	
108	What is partner'soccupation status?	1= Farmer<br>2=Self-employed<br>3=Merchant<br>4=Governmental employed<br>5=Daily laborer<br>99= Others<br>specify……………….	
109	Place of residence?	1= Rural<br>2=Urban	
110	Type of study participants under interview?	1=Host<br>2=Internal displaced people	
111	Do you have a radio/television in your household?	1=Yes<br>2=No	
112	Do you listen to the radio or watch television?	1=Always/every day<br>2=At least once a week<br>3= Less than once a week<br>4= Not at all	

113	Family size?	………………in number	
**Reproductive and maternal health**			
201	How many of pregnancies have you conceived up to date?	______ (in number)	
202	How many of births have you gave up to date?	______ (in number)	
203	How many live births do you have up to now?	______ (in number)	
204	How many still births do you have up to now?	______ (in number)	
205	How many abortions did face in your life time?	______ (in number)	
206	have you had ANC follow-ups during your last child?	1=Yes<br>2=No	
207	If yes to Q 206, how many times did you receive antenatal care for your pregnancy?	Number of times------------	
208	If yes to Q 206, at what gestational age did you first attend ANC for the current/last pregnancy?	-----------week<br><br>98= Don't know	
209	If no to Q 206, what was the main reason for not had ANC follow-up?	1=Health facility was closed<br>2=No health worker at the facility<br>3=No supply and medicine for ANC<br>4=Security concern<br>5=Displacement<br>6= No public transport<br>7=Other, Specify___	
210	Did you take Iron folate tablets during previous pregnancies?	1=Yes<br>2=No	

ማሕበራዊን ስነህዝባዊን ነታት			
ተ.ቑ	ሕቶ	ምላሽ	ሽገር
101	ዕድመቃለ-መሕተት ዝተገበረሉ/ላ	…………… (ብዓመት)	
102	ኩነታትሓዳር	1=ዘይተመርዐወ/ት<br>2=ዝተመርዐወ/ት<br>3=ዝተፋተሐት/ሐ<br>4=ዝሞታ/ዝሞተቶ<br>5=ብሓደዘይትነብር/ዘይነብር	
103	ኩነታትትምህርቲ	1= ምንባብን ምፅሓፍን ዘይትኽእል/ዘይኽእል<br>2=ምንባብን ምፅሓፍን ትኽእል/ዝኽእል ግን ስሩዕትምህርቲ ዘይኣተወት/ዘይኣተወ<br>3=ቀዳማይ ብርኪትምህርቲ<br>4= ካልኣይ ብርኪ ትምህርቲ<br>5= ኮሌጅን ልዕሊኡን	
104	ኩነታትትምህርቲበዓልገዛኺ/ገዛኻ	1= ምንባብን ምፅሓፍንቨ ዘይትኽእል/ዘይኽእል<br>2=ምንባብን ምፅሓፍን ትኽእል/ዝኽእል ግን ስሩዕ ትምህርቲ ዘይኣተወት/ዘይኣተወ<br>3=ቀዳማይ ብርኪ ትምህርቲ<br>4= ካልኣይ ብርኪ ትምህርቲ<br>5= ኮሌጅንመ ልዕሊኡን	
105	ትኽተልዮ/ተሎሃይማኖት	1=ኦርቶዶክስ<br>2=ፕሮቲስታንት<br>3=ሙስሊም<br>4=ካቶሊክ<br>99=ካሊእይገለፅ……….	
106	ብሄር	1= ትግራዋይ/ቲ<br>2= ኢሮብ<br>3= ዓፋር<br>4=ኣምሓራ<br>99= ካሊእ እንተኾይኑ ይገለፅ………..	
107		1=ኣብገዝኣትስርሕ	

	መደብ ስራሕኺ	2=ኣብ ናይ ውልቀ ትካል ቁፃር<br>3=ነጋዲት<br>4=መንግስታዊ ቁፃር<br>5=መዓልታዊ ሰራሕተኛ<br>6= ሓረስታይ<br>99=ካሊእይገለፅ…….	
108	መደብ ስራሕ በዓልገ ዛኺ	1= ሓረስታይ<br>2=ኣብ ናይ ውልቀ ትካል ቁፃር<br>3=ነጋዲይ<br>4=መንግስታዊ ቁፃር<br>5=መዓልታዊ ሰራሕተኛ<br>99=ካሊእ ይገለፅ………………………	
109	ትነብርሉ/ትነብረሉ ከባቢ/ቦታ<br><br>***መብርሂ፥ ሓውሲ-ከተማዝብልንከተሚታትገጠርወረ ዳዝተወሃበስምእዩ***	1= ገጠር<br>2=ሓውሲ-ከተማ<br>3=ከተማ	
110	ኩነታት ሓበሬታ ዝሃበ ተሳታፋይ	1=ቀዋሚነባራይ<br>2=ተመዛባላይ	
111	ኣብ ገዛኹ ምራድዮ ወይ ቴሌቪዥን ኣለኩም ዶ?	1=እወ<br>2=ኣይፋል	
112	መዓዝመዓዝትሰምዕ/ዒ ወይ ትርኢ?	1. ኩሉ ግዘ/በቢመዓል<br>2. እነተነኣሰ ኣብ ሰሙን ሓደ ግዘ<br>3. ካብ ሓደ ግዘዝነኣሰ ኣብ ሰሙን<br>4. ምንም ኣይርእን	
113	በዝሒ ስድራ	**………………(ብቑፅሪ)**	
**ኣብ ግልጋሎት ጥዕና ኣዴታትን ስነ-ተዋልዶ ን ዝተድረኹ ሕቶታት**			
201	ክሳብ ሐዚ ንክንደይ ግዜ ጠኒስኪ?	_______ (ብ ቁፅሪ)	
202	ክሳብ ሐዚ ብጠቕላላ ክንደይ ቆልዑ ወሊድኪ?	_______ (ብ ቁፅሪ)	
203	ካብዚኦም ክንደይ ቆልዑ ምስሂወቶም ውሊድኪ?	_______ (ብ ቁፅሪ)	
204	ካብዚኦም ክንደይ ቆልዓ/ዑ ምውት/ታት ወሊድኪ?	_______ (ብ ቁፅሪ)	
205	ክሳብ ሐዚ ክንደይ ግዜ ምንፃል ጥንሲ ኣጋጢሙኪ?	_______ (ብ ቁፅሪ)	

206	ኣብ ግዜ ጥንስኪ ናይ ቅድመ ወሊድ ክትትል ገይርኺ ዶ?	1=እወ<br>2=ኣይፋል	
207	ን ሕቶ ቁፅሪ 206 እወ እንተኾይኑ ንኽንደይ ግዜ ናይ ቅድመ ወሊድ ክትትል ጌርኪ?	ብ ቁፅሪ ይገለፅ_______	
208	ን ሕቶ ቁፅሪ 206 እወ እንተኾይኑ ፈላማይ ናይ ቅድመ ወሊድ ክትትል ዝረኸብክሉ ኣብ ክንደይ ሰሙን ነይሩ?	____ ሰሙን<br>98 = ኣይፈልጥን	
209	ን ሕቶ ቁፅሪ 206 ኣይፋል እንተኾይኑ ቀንዲ ምኽንያት ቅድመ ወሊድ ክትትል ዘይምግባረኪ እንታይ ነይሩ?	1= ጥዕና ትካል ስለ ዝተዐፀወ<br>2=ሰብ ሞያ ጥዕና ኣብ ጥዕና ትካል ስለዘይነበሩ<br>3=~~ናይ~~ ቀረብ መድሓኒትን ካላኦት ሕክምና ናውትን ስለዘይነበረ<br>4=ናይ ድሕንነት ስግኣት ስለዝነበረ<br>5= ስለ ዝተፈናቐልኩ<br>6= መጓዓዝያ ስለዘይነበረ<br>7= ካልእ ይጠቐስ________	
210	ኣብ እዋን ጥንስኪ አይረንእናፎሊክ አሲድ ዝሓዘ ንጥረ ነገር ዝሓዘ ወሲድኪ ኔርኪ ዶ	1=እወ<br>2=ኣይፋል	
