# Supplementary material for: Effective coverage of antenatal care services in post war Tigray, Northern Ethiopia: An analysis of community and health facility–based surveys
Source: PLoS One. 2025 Oct 30;20(10):e0336121. doi: 10.1371/journal.pone.0336121 (PMC12574916; doi:10.1371/journal.pone.0336121)
Supplement: S2 File — (PDF) [file pone.0336121.s002.pdf]

A Checklist for assessment of *antenatal care services* readiness across health facility level in Tigray

Name of District ______________________

Name of Health Facility_____________________________

Health Facility type ____________________________

Total catchment population_____________________

Date of data collection: _________________

Equipment and diagnostic capacity

**Items for providing ANC. For each item I ask about please tell me if it is available and functional and then show it to me.**			
List of tracer items	Yes, observed	Yes, reported not seen	No
Access to emergency transport	1	2	0
Blood pressure apparatus	1	2	0
Foetal stethoscope/pinard/digital	1	2	0
Adult weighing scale	1	2	0
Examination bed	1	2	0
Tape measure	1	2	0
ITN	1	2	0
Hemoglobin test	1	2	0
Urine protein test	1	2	0
Syphilis test	1	2	0
HIV test ki	1	2	0
Iron-folate tablets	1	2	0
Tetanus toxoid vaccines	1	2	0

Availability ANC guidelines and trained staffs

Please tell me if the following documents are available in the facility today: If available, ask to see the document.	Yes, observed	Yes, reported not seen	No
National ANC guidelines	1	2	0
Any ANC check-lists and/or job-aids	1	2	0
National guidelines on Intermittent preventive treatment in pregnancy (IPTp) for malaria	1	2	0
IPTp checklists and/or job aids (including wall charts)	1	2	0
**In the past 2 years, have you or any provider(s) of ANC received training in any of the following topics:**	**YES**	**NO**	
Any aspect of ANC	1	0	
IPTp for malaria	1	0	
