# Supplementary material for: Effective coverage of antenatal care services in post war Tigray, Northern Ethiopia: An analysis of community and health facility–based surveys
Source: PLoS One. 2025 Oct 30;20(10):e0336121. doi: 10.1371/journal.pone.0336121 (PMC12574916; doi:10.1371/journal.pone.0336121)
Supplement: S3 File — (PDF) [file pone.0336121.s003.pdf]

An observation Checklist to assess process quality of *antenatal care services*

Zone ________________________________

Woreda______________________________

Name of Health Facility_____________________________

Health Facility type ____________________________

Total catchment population____________________

Date of data collection: _________________

ANTENATAL CARE SERVICES	***Yes***	***No***	***Remark***
**Observe the provider for the following indicators while S/he provide ANC:**<br>Review the history, LMP/ calculate GA/EDD	1	0	
Ask about fetal movement	1	0	
Enquire about any complaint or concern	1	0	
Monitoring for hypertensive disorder of pregnancy (measure blood pressure)	1	0	
Measure weight check for weight gain	1	0	
Look for pallor	1	0	
Measure arm for acute malnutrition screening using MUAC	1	0	
Measure the uterine fundal height	1	0	
Listen for fetal heartbeat	1	0	
Initiate iron-folate and calcium supplementation and counsel on adherence	1	0	
Check & told about the danger signs and symptoms of pregnancy (bleeding, severe head ache, blurred vision, loos of fetal movement, and abdominal pain…)	1	0	
Assess feeding practices and counsel on optimal maternal nutrition	1	0	
Provide Preventive chemotherapy (deworming)	1	0	
Does the mother test for the following:<br>1. HGB/HCt	1	0	
2. Urine analysis (check urine protein)	1	0	
3. Syphilis	1	0	
4. HIV and counseling	1	0	
