# Supplementary material for: Effective coverage of antenatal care services in post war Tigray, Northern Ethiopia: An analysis of community and health facility–based surveys
Source: PLoS One. 2025 Oct 30;20(10):e0336121. doi: 10.1371/journal.pone.0336121 (PMC12574916; doi:10.1371/journal.pone.0336121)
Supplement: S4 File — (DOCX) [file pone.0336121.s004.docx]

STROBE Statement—Checklist of items that should be included in reports of **cross-sectional studies**

Study Title: **Effective coverage of antenatal care services in post war Tigray, Northern Ethiopia: An analysis of community and health facility-based surveys**

|  | Item No | Recommendation |
| --- | --- | --- |
| **Title and abstract** | 1 | (*a*) Indicate the study’s design with a commonly used term in the title or the abstract  In the abstract: page 2; line 38. |
|  |  | (*b*) Provide in the abstract an informative and balanced summary of what was done and what was found Abstract: Page 2; line from 38-52. |
| Introduction | | |
| Background/rationale | 2 | Explain the scientific background and rationale for the investigation being reported Introduction: page 4and 5; and line 93-102. |
| Objectives | 3 | State specific objectives, including any prespecified hypotheses Introduction: page 5; line 101-102. |
| Methods | | |
| Study design | 4 | Present key elements of study design early in the paper Methods (on study design, setting sand period sub section) page 4; line 105. |
| Setting | 5 | Describe the setting, locations, and relevant dates, including periods of recruitment, exposure, follow-up, and data collection Methods (on study design and setting sub section) page 5; line 105-111. |
| Participants | 6 | (*a*) Give the eligibility criteria, and the sources and methods of selection of participants Methods (Study population sub section) page 5 and 6; line 113-116. |
| Variables | 7 | Clearly define all outcomes, exposures, predictors, potential confounders, and effect modifiers. Give diagnostic criteria, if applicable Methods (Measurement of variables  sub section) page 8,9; line 162-195. |
| Data sources/measurement | 8* | For each variable of interest, give sources of data and details of methods of assessment (measurement). Describe comparability of assessment methods if there is more than one group Methods (Data collection tools and techniques sub section) page 6 and 7; line 137-150. |
| Bias | 9 | Describe any efforts to address potential sources of bias Methods (data quality assurances sub section) page 7; line 152-160. |
| Study size | 10 | Explain how the study size was arrived at Methods (sample size and sampling procedure sub section) page 5and 6; line 121-125. |
| Quantitative variables | 11 | Explain how quantitative variables were handled in the analyses. If applicable, describe which groupings were chosen and why Methods (data management and analysis sub sectio page 7; line 144-145 |
| Statistical methods | 12 | (*a*) Describe all statistical methods, including those used to control for confounding Methods (data management and analysis sub section) page 9; line 197-211. |
|  |  | (*b*) Describe any methods used to examine subgroups and interactions |
|  |  | (*c*) Explain how missing data were addressed |
|  |  | (*d*) If applicable, describe analytical methods taking account of sampling strategy |
|  |  | (*e*) Describe any sensitivity analyses |
| Results | | |
| Participants | 13* | (a) Report numbers of individuals at each stage of study—eg numbers potentially eligible, examined for eligibility, confirmed eligible, included in the study, completing follow-up, and analysed Results page 10; line 223-226. |
|  |  | (b) Give reasons for non-participation at each stage |
|  |  | (c) Consider use of a flow diagram |
| Descriptive data | 14* | (a) Give characteristics of study participants (eg demographic, clinical, social) and information on exposures and potential confounders Results page 10-12; line 227-240. |
|  |  | (b) Indicate number of participants with missing data for each variable of interest |
| Outcome data | 15* | Report numbers of outcome events or summary measures Results page 12-15; line 246-274. |
| Main results | 16 | (*a*) Give unadjusted estimates and, if applicable, confounder-adjusted estimates and their precision (eg, 95% confidence interval). Make clear which confounders were adjusted for and why they were included. |
|  |  | (*b*) Report category boundaries when continuous variables were categorized |
|  |  | (*c*) If relevant, consider translating estimates of relative risk into absolute risk for a meaningful time period |
| Other analyses | 17 | Report other analyses done—eg analyses of subgroups and interactions, and sensitivity analyses |
| Discussion | | |
| Key results | 18 | Summarise key results with reference to study objectives Discussion page 15; line 276-280. |
| Limitations | 19 | Discuss limitations of the study, taking into account sources of potential bias or imprecision. Discuss both direction and magnitude of any potential bias Discussion (strength and limitation sub section) page 18; line 343-349. |
| Interpretation | 20 | Give a cautious overall interpretation of results considering objectives, limitations, multiplicity of analyses, results from similar studies, and other relevant evidence Conclusion page 19; lines 351-360. |
| Generalisability | 21 | Discuss the generalisability (external validity) of the study results bias Discussion 8(strength and limitation sub section) page 18; line 344-345. |
| Other information | | |
| Funding | 22 | Give the source of funding and the role of the funders for the present study and, if applicable, for the original study on which the present article is based  The study was financially supported by Tigray Health Bureau. Its contents are solely the responsibility of the authors and do not necessarily represent the official views of the supporting offices. The funder had no role in study design, data collection and analysis, decision to publish or preparation of the manuscript*.* |

*Give information separately for exposed and unexposed groups.

**Note:** An Explanation and Elaboration article discusses each checklist item and gives methodological background and published examples of transparent reporting. The STROBE checklist is best used in conjunction with this article (freely available on the Web sites of PLoS Medicine at http://www.plosmedicine.org/, Annals of Internal Medicine at http://www.annals.org/, and Epidemiology at http://www.epidem.com/). Information on the STROBE Initiative is available at www.strobe-statement.org.
