# Supplementary material for: Effective coverage of antenatal care services in post war Tigray, Northern Ethiopia: An analysis of community and health facility–based surveys
Source: PLoS One. 2025 Oct 30;20(10):e0336121. doi: 10.1371/journal.pone.0336121 (PMC12574916; doi:10.1371/journal.pone.0336121)
Supplement: Attachment — Submitted filename: Response to Reviewers.docx [file pone.0336121.s007.docx]

**Responses to editor’s and reviewers’ comments**

**Editor ’s comment**

**Journal Requirements:**

**Editor ’s comment**

1. Please ensure that your manuscript meets PLOS ONE's style requirements, including those for file naming. The PLOS ONE style templates can be found at

**Response to editor’s comment:** Thank you for your guidance, we have tried to revise our manuscript based on the PLOSE ONE journal style template and journal requirements.

**Editor ’s comment**

2. We note that the grant information you provided in the ‘Funding Information’ and ‘Financial Disclosure’ sections do not match.

**Response to editor’s comment:** Thank you for your insightful comment. However, the study was supported by per diem and vehicle support provided by Tigray Health Bureau. There is no formal grant number associated with this support.
**Additional Editor Comments:**
**Editor ’s comment:** The paper requires drastic improvement for English language. There are multiple issues such as typos, grammatic errors, word usage, and formatting. I recommend you seek proof reading from proficient individual.
**Response to editor’s comment:** We are grateful for your comment. We have made significant language corrections as advised.

**Background**
**Editor ’s comment:** The entire background needs to be rewritten, the AI score is 90%.
Please improve the flow of the background,
**Response to editor’s comment:** Thank you, we have rewritten the background as advised.

**Editor ’s comment:** Lines 68-73 have complementary ideas but you have tried to connect them as if they were conflicting.
**Response to editor’s comment:** Thank you, we have revised the paragraph accordingly.

**Editor ’s comment:** Please address this in your introductions. How can health care providers and policy makers effectively mitigate the challenges in resource and quality of care in the region. What strategies have been implemented to improve effective ANC coverage in war affected areas like Tigray.
**Response to editor’s comment:** Thank you for your insightful comment**.** We have added as suggested.

The revised introduction section on page 5, paragraph 6 and line 99-103 now reads, “Following the signing of the Pretoria cessation of hostility agreement on November 2, 2022[27], the Tigray Regional Health Bureau in collaboration with local and international partners, launched health recovery initiatives to rebuild the war-affected health system of Tigray. These efforts include supplying medical equipment and furniture, providing outreach health services, enhancing the capacity of healthcare providers and strengthening the overall health system.”

**Methods**
**Editor ’s comment:** You are advised to cite the source for the information regarding population data. For instance, Line 108-111.
**Response to editor’s comment:** Thank you for your comment. I have added a reference as suggested.
**Editor ’s comment:** Please cite the study, or if you are unable to do so, remove the statements 118-120.
**Response to editor’s comment:** Thank you for your comment. We added a reference as suggested.

**Editor ’s comment:** The sample size and sampling procedure is difficult to decipher for the readers. Schematic presentation, i.e figure, would make it easier. Furthermore, you have to explain why you selected 8 urban/16 rural. Why 30% Tabias, and why 30 women. In the first stage, a total of 24 districts (8 urban and 16 rural) were randomly selected from 78 accessible districts of Tigray. The second stage, 30% Tabias were selected randomly (a total of 78 tabias) from the 24 selected districts. Finally, 30 women with under one year child were randomly selected from each selected Tabia’

**Response to editor’s comment:** Thank you, we have added Schematic presentation by figure in the revised manuscript as suggested.

Regarding the selection districts, Tabias and eligible women. We did not intentionally select 8 urban and 16 rural. We randomly selected 24 districts from the 76 accessible districts, which resulted in a selection of 8 urban and 16 rural districts by chance. Regarding our selection of 30% of Tabias and 30 women with children under one year old, we recognize that including the entire population or a large sample size is ideal for representativeness. However, due to resource constraints, this approach is not feasible. Therefore, selecting 30% of the sample size is a feasible that balances representativeness with practicality in our research design. Additionally, we treated each Tabia as a cluster and included 30 households per Tabia/cluster due to these limited resources. As we have detailed in the sample size and sampling procedure section, selecting 20-30 households per enumeration area/cluster is considered adequate for our study [27].

**Editor ’s comment:** Why 32 primary hospitals and what is the basis for the proportion of 9 primary hospitals vs 23 health center. How did you come up with the 250 ANC clients, and the proportion of 5 to 15 ? Justify it/ cite reference.
**Response to editor’s comment:** Our objective is to link the community-based study with a health facility-based study to evaluate the effective coverage of antenatal care (ANC) services. To achieve this, we included all 32 primary health care facilities (9 primary hospitals and 23 health centers) that serve the selected Tabias to assess health facility readiness. Additionally, we purposively sampled 250 ANC clients based on expert consensus and then we selected an average of 5 clients from each health center and 15 clients from each primary hospital by systematic random sampling by considering the ANC client load from the previous three months to assess ANC quality process components.

**Editor ’s comment:** ANC 4, It would help if you mentioned it as a limitation, why most of your metrics fall within the old ANC recommendations of 4 visits instead of contacts of the revised WHO recommendations for positive pregnancy experience.
**Response to editor’s comment:** Thank you for your comment. However, the revised WHO recommendations, which suggest at least 8 contacts during pregnancy have not yet been fully implemented in the Tigray region due to the conflict. Furthermore, effective coverage of ANC services can be calculated without requiring ANC4 or ANC8; it can also be determined using the coverage of ANC1 (26).

**Discussion
Editor ’s comment:** The discussion needs a massive upgrade. It is important to showcase the importance of the study more precisely. Currently, the argument utilized in the discussion is shallow/superficial. A more detailed comparison with pre war conditions is important. Furthermore, please compare it with other conflict ridden areas. Most importantly, emphasize on showcasing how the impact of the Tigray war varies from the effects of other wars on ANC utilization and ANC quality. Please factor in the dynamics of the war in this context.
**Response to editor’s comment:** Thank you, we have done major modification in discussion section as suggested in the revised manuscript.

**Conclusions
Editor ’s comment:** You have to stick to your study findings. Some of the recommendations put forward are from preexisting knowledge that predates your findings.
What is the implication of your study?
**Response to editor’s comment:** Thank you for your comment. We have aligned our recommendations based on our findings as suggested.
**Reviewers' comments:**

**Reviewer #1**

**Methods**
**Reviewer’s comment:** This study uses multistage survey data. The authors employed a multistage cluster sampling technique to select districts, tabiyas, and households. Typically, multistage surveys require sample weighting to account for unequal probabilities of selection. Based on the selection criteria described, not all households had an equal chance of being included in the study. Applying sample weighting would adjust for this unequal probability and improve the representativeness of the findings. If feasible, I recommend incorporating sample weighting to make the results more reflective of the target population.
**Response to review’s comment:** Thank for your observation. Yes, of course, it recommended to use sapling weight in multistage cluster sampling design, However, it not always strict requirements because in our case we assume that the study population had qual probabilities of being selected and the same characteristics. In our sampling strategy, all individuals or households within the sampled Tabias were selected with equal probability using simple random technique. As such, there was no unequal selection probability to adjust for, eliminating the need for sample weights.

**Results
Reviewer’s comment:** Page 12, line 242: The subtitle reads, “Reasons for not had ANC follow-up.” Please revise this to correct the grammar. Additionally, ensure consistency in language usage throughout the manuscript. For example, while both "centre" and "center" are correct spellings, they belong to British and American English, respectively. Consistency in style is recommended.
**Response to reviewer’s comment:** Thank you, the comment is well accepted. We have edited the “Reasons for not had ANC follow-up” to “Reasons for not attending ANC follow-up” and we changed word “centre" to “center" in revised manuscript.
**Discussion
Reviewer’s comment:** The study was conducted in early 2024, at least a year after the war ended. An ANC coverage of 87.4% is not particularly low and warrants further explanation in the discussion. For example, what was the ANC1 coverage during the war (very likely it was much lower than 87.4%)? What actions were taken in the one-year post-war period that led to this coverage to 87.4% level? Although it is lower than the pre-war period, it is still relatively high, and understanding these dynamics would enhance the discussion.

**Response to reviewer’s comment:** Thank you for your insightful comment. We have incorporated the comment in the revised manuscript as suggested. The revised discussion section on page 17, paragraph 4 and line 307-313 now reads,” On the other hand, ANC1 coverage (87.4%) in this study was higher compared to the period during the war (54.9%) [53]. This increase could be attributed to the implementation of health recovery initiatives following the Pretoria Cessation of Hostilities Agreement. Health facilities have resumed providing antenatal care (ANC) services, which may have encouraged greater community health–seeking behaviour toward ANC services. However, this observation requires careful interpretation, as attending ANC visit does not necessarily indicate that client received all the essential components of ANC services during their visits”.

**Strengths and Limitations
Reviewer’s comment:** The main limitation of this study is that it did not adjust for potential confounders, which may have contributed to the observed lower ANC coverage during the post-war period. This limitation should be clearly mentioned.

**Response to reviewer’s comment**: Thank you for your comment. However, our primary objective in this study is to assess the effective coverage of antenatal care (ANC) services. Identifying factors that contribute to lower ANC coverage is not our objective. Effective coverage is a relatively new concept defined as the proportion of a population in need of a service that gains a positive health outcome from that service (5). To calculate the effective coverage of ANC, we simply multiplied the proportion of women who received four or more ANC visits by the mean facility readiness score, the proportion of women who received iron-folate, and the average process quality score (6).

**Conclusions
Reviewer’s comment:** Page 19, line 352: The conclusion is not fully supported by the findings. The authors state that inadequate facility readiness and suboptimal quality of care contributed to the lower ANC coverage, but no analysis was conducted to support this claim. I suggest revising the conclusion to align with the results.

**Response to reviewer’s comment:** Thank you for your comment. It is important to clarify that our conclusion pertains to the low effective coverage of antenatal care (ANC) services, which is different from the low ANC coverage. Normally, we could not identify the factors affecting for low effective coverage of ANC services by statistical analysis using bivariate and multi-variable model analysis. However, as we have clearly stated in method section effective coverage of ANC services was calculated by multiplying the proportion of women who received four or more ANC visits (15.7%) by the mean of health facility readiness score (55.6%), the proportion women who received iron-folate (81.4%), and the average process quality score (53.8%).

I.e. effective coverage of ANC services=15.7%*55.6%*81.4%*53.8%= (0.157*0.556*0.814*0.538) * 100% =3.8%.

The above calculation indicates that effective coverage of ANC services is affected by coverage of ANC4+, mean of health facility readiness score, coverage of iron-folate supplementation, and ANC basic services process quality score.

- ANC1 coverage =87.4% and ANC4+=15.7%. Therefore, there is a significant drop-off in continuity of care from the first to the fourth ANC visit (from 87.4% to15.7%.).
- Mean of health facility readiness score=55.6% it indicates inadequate facility readiness.
- Mean of basic ANC quality process score=53.8% it indicates suboptimal quality of ANC service.

Therefore, with the above justification, we believe that our conclusion is based on our findings. “The overall effective coverage of ANC services in post war Tigray is very low, with deficiencies observed across each component of the cascade. The primary factors contributing to this low effective coverage of ANC services include a significant drop-off in continuity of care from the first to the fourth ANC visit, as well as inadequate facility readiness and suboptimal quality of care service”.

**Reviewer #2**

**Reviewer’s comment:** • As the WHO currently revise the schedule for 8 ANC visit, but the author uses 4 visits? What is your justification.

**Response to reviewer’s comment:** Thank you for your comment. However, the revised WHO recommendations, which suggest at least 8 contacts during pregnancy have not yet been fully implemented in the Tigray region due to the conflict. Furthermore, effective coverage of ANC services can be calculated without requiring ANC4 or ANC8; it can also be determined using the coverage of ANC1 (26).
**Reviewer’s comment:** • As this study was done in a post recovery from the war, dealing on quality could be matter. However, it was cognizant that 70% of the health facilities were damaged or looted?
**Response to reviewer’s comment:** Thank you for your comment. As we mentioned in the methods section, this study is part of the large study which was included the services utilization and effective coverage because promoting high-quality care is particularly important in post-conflict settings (8).
**Abstract
Reviewer’s comment:** • On the background section, the authors described that … Although promoting high-quality care is particularly important in post-conflict settings, little is known. However, in post conflict setting despite the quality of the service matter but the priority should be to enhance access for the service as the service was dramatically damaged due to the war.
• In line with this, what is the real gap for conducting such study should be described in a brief manner.
**Response to reviewer’s comment:** Thank you for your insightful comment. Absolutely true, access to services is a top priority in post-war settings. However, in our assessment of effective coverage, we also evaluated service access and contact coverage. Access to health services/ health facility readiness is key components of effective coverage.

**Reviewer’s comment:** • On the method section, there are two populations
o 2340 mothers of children under one year
o 250 antenatal care (ANC) clients from the selected health facilities were included in
♣ What is the difference between these two populations?

**Response to reviewer’s comment:** Our objective is to link the community-based study with a health facility-based study to evaluate the effective coverage of antenatal care (ANC) services. To achieve this, we included 2340 mothers of children under one year to assess crude ANC coverage (coverage of ANC+), we included 32 primary health care facilities to assess the health facility readiness, and we included 250 antenatal care (ANC) clients from the selected health facilities to evaluate the ANC process quality.

**Reviewer’s comment:** o Add the type of analysis employed to answer the objective of the study

**Response to reviewer’s comment:** Thank you for your feedback. As suggested, we have included details about our analysis in the revised manuscript.

**Reviewer’s comment:** o The research put an output for the Input-and intervention-adjusted ANC and what about for the contact coverage and Quality adjusted coverage related to the variable?

**Response to reviewer’s comment:** Thank you for your comment. As we mentioned in the methods section (Measurement of Variables sub- subsection) on page 8, lines 168 and 169, contact coverage refers to the proportion of women who attended four or more ANC visits. Accordingly, we reported ANC+ coverage in our findings. Regarding quality-adjusted coverage, we used it as a proxy for effective coverage because data on client adherence and outcome-adjusted coverage were unavailable, as recommended by the "Effective Coverage Think Tank Group" [5]. In our study, quality-adjusted coverage is equivalent to effective coverage. This is clearly detailed in the Measurement of Variables subsection.

**Results
Reviewer’s comment:** • Do you have quality-adjusted antenatal coverage estimates?
**Response to reviewer’s comment:** In our study, quality-adjusted antenatal coverage is equivalent to effective coverage of ANC services. We took quality adjusted ANC coverage as a proxy measure of effective coverage, as we don’t have information on the other two components of the effective coverage cascade (client adherence and outcome adjusted coverage) as recommended by the “Effective coverage Think Tank Group” (5).

**Introduction
Reviewer’s comment:** • Overall, it was organized very well. However, what could be the justification for emphasising on quality than in access in area where the 70% of the health facilities were not functional or partially functional.
**Response to reviewer’s comment:** Thank you for your insightful comment. Absolutely true, access to services is a top priority in post-war settings. However, in our assessment of effective coverage, we also evaluated service access and contact coverage. Access to health services/ health facility readiness is key components of effective coverage. Therefore, we had also assessed the access to health services.

**Methods
Reviewer’s comment:** The authors described that this study was part of a large study. Does the large study published? If yes add the reference rather than describing a sampling procedure of the Ethiopia demographic health survey

**Response to reviewer’s comment:** Thank you, a large study is not published, but we have included it as an unpublished reference.

**Reviewer’s comment:** • Do you have a sampling frame to select the eligible women as what they did in the EDHS?

**Response to reviewer’s comment:** Yes, we used a sampling frame of the eligible women from HEWs.

**Reviewer’s comment:** • Does the tool part of the large study or you develop a tool specifically for this study?

**Response to reviewer’s comment:** The tool was part of the large study; it had common section on socio-demographic information, and it included maternal health services as utilization section. **Reviewer’s comment:** • On the measurement section to describe the variable of interest under your investigation?

o Intervention coverage. This refers to the proportion of women who received iron-folate tablets.? what about TT vaccination

**Response to reviewer’s comment:** Thank you for your comment. Effective coverage of antenatal care (ANC) services can be assessed by selecting one or more interventions (5). Alternatively, it can be calculated without selecting a particular intervention by multiplying the average quality of service by crude or contact coverage (26).

**Results
Reviewer’s comment:** • Can you organize the table 2 and 3 information using graph?

**Response to reviewer’s comment:** Thank you for your insightful comment. Yes of course, graphs offer better visualization compared to tables. However, our focus is on comparing the availability of ANC tracer items and the process quality of ANC across health centers and primary hospitals side by side of the total frequency and percentage.

**Discussion
Reviewer’s comment:** • How conducting a study in community and health facility setting which increased its reliability.

**Response to reviewer’s comment:** Conducting a study in both community and health facility settings can increase its reliability by ensuring diverse data sources and different data collection techniques like; interviews, observations and document review.

**Reviewer’s comment:** • What mechanism does the authors uses to reduce Howerton effect bias? What type of observation, does the authors use to observe for the process quality?
**Response to reviewer’s comment:** Thank you; we used direct observation to assess the process quality of ANC services. To reduce the Hawthorne effect and observer bias during our direct observation, we implemented several strategies including:

- We used standardized checklists to ensure uniform data collection across all observations, reducing variability and enhancing consistency.
- Our data collectors underwent intensive training on study purpose, ethical considerations, how to minimize observer bias and how to ensure consistency during the observation.

**Reviewer’s comment:** The exclusion of some districts as result of security issue mentioned as limitation of the study. Is that real limitation?

**Response to reviewer’s comment:** Thank you for your comment. It is likely that the actual effective ANC coverage is lower than the estimates reported in this study, as certain districts were excluded due to security concerns. These excluded districts may have experienced significant damage to health facilities and reduced ANC contact coverage, further affecting the effective delivery of ANC services. This limitation should be reported in the study's limitations section to ensure that future users of these findings take this factor into account.

**Reviewer’s comment:** o Do you have methodological limitation like not including TT vaccination in the measurement of intervention coverage.
**Response to reviewer’s comment:** Thank you for your comment. Effective coverage of antenatal care (ANC) services can be assessed by selecting one or more interventions (5). Alternatively, it can be calculated without selecting a particular intervention by multiplying the average quality of service by crude or contact coverage (26). Therefore, not including TT vaccination in our study is not limitation.

**Conclusion
Reviewer’s comment:** • The first paragraph…. You conclude that he overall effective coverage of ANC services in post war Tigray is very low
o It was obvious as the study was done in post conflict setting.
**Response to reviewer’s comment:** Thank you for your comment. Although it is obvious that effective ANC coverage tends to decline in post-war periods, the reduction observed in this study is drastic, showing a tenfold decrease compared to the pre-war period. The coverage declined significantly from 38.7% before the war to just 3.8% in this study (26).
