# Supplementary material for: Effective coverage of antenatal care services in post war Tigray, Northern Ethiopia: An analysis of community and health facility–based surveys
Source: PLoS One. 2025 Oct 30;20(10):e0336121. doi: 10.1371/journal.pone.0336121 (PMC12574916; doi:10.1371/journal.pone.0336121)
Supplement: Attachment — Submitted filename: comments to the Authors.docx [file pone.0336121.s006.docx]

Dear editor,

I would like to thank the editor for the opportunity to review this interesting manuscript. After carefully evaluating the study, I find that it has merit; however, I recommend the following revisions to enhance its clarity and rigor.

Comments and Suggestions for the Authors:

1. Sampling Technique: The methodology regarding participant selection is not clearly described. The authors should provide a detailed explanation of the sampling process and illustrate the number of participants recruited from each district using a diagram.
2. Data Quality Assurance: The manuscript states that the questionnaire underwent a pre-test in a different location to assess response accuracy and estimate the required time. Please specify the location where the pre-test was conducted and describe any adjustments made based on the pre-test results.
3. The results are well-articulated and clearly presented.
4. Given the reported low effective coverage of antenatal care (ANC) services in post-war Tigray, it is important to specify the baseline used for comparison. Clarifying this will help contextualize the findings.
5. Strengths and Limitations: The section discussing the strengths and limitations of the study is not well-developed. A thorough revision is needed to clearly highlight the key strengths and potential limitations.
6. It would be beneficial for the authors to suggest directions for future research based on the study’s findings.
7. The conclusion does not fully reflect the study’s key findings. A thorough revision is needed to ensure alignment with the results and key discussions.
8. References: DOIs should be included for all referenced articles where applicable
