# Supplementary material for: Effective coverage of antenatal care services in post war Tigray, Northern Ethiopia: An analysis of community and health facility–based surveys
Source: PLoS One. 2025 Oct 30;20(10):e0336121. doi: 10.1371/journal.pone.0336121 (PMC12574916; doi:10.1371/journal.pone.0336121)
Supplement: Attachment — Submitted filename: Responses to reviewers.docx [file pone.0336121.s008.docx]

**Editor’s comments:**

**1. Introduction**

**Editor ’s comment:** The introduction provides adequate background on the importance of ANC and the challenges in post-conflict settings. However, the emphasis on quality over access in the introduction may seem contradictory given the context where 70% of health facilities were damaged or looted. Clarify how the study aims to balance these priorities.

**Response to editor’s comment:** Thank you for your comment. You are right, ensuring access to ANC services is a top priority in post-war environments, where 70% of health facilities have been damaged or looted. However, it is equally important to promote high-quality care in these post-conflict settings (8). In our assessment, we incorporated various dimensions to evaluate effective coverage, including access to health services/health facility readiness, contact coverage, intervention coverage, and quality processes. Since the readiness of health facilities is a key component of effective coverage, we believe our study adequately addresses the access to ANC services as well.

**Editor ’s comment:** Ensure the research gap is clearly articulated. For example, why is effective coverage of ANC particularly relevant in this context compared to contact coverage or access alone?

**Response to editor’s comment:** Thank you for your insightful comment. However, contact coverage or access is nothing without high quality of care. Additionally, the components of effective coverage are facility readiness, contact coverage, intervention coverage and process quality. Therefore, we have assessed effective coverage and all its components as the time.

**2. Methods**

**Editor ’s comment:** The methods section is detailed but could benefit from more organization. For instance, separating sampling procedures for the community based and health facility-based components into distinct subsections would improve readability.

**Response to editor’s comment:** Thank you for your valuable comment. In our study, the community-based and health facility-based components are linked through the sampling procedures, as the chosen health facilities are located within the selected Tabias that serve the catchment population.

**Editor ’s comment:** The tools adapted from EDHS and WHO should be briefly described.

**Response to editor’s comment:** Thank you for your insightful comment**.** We have described as suggested.

The revised methods section (data collection tools and techniques sub-section) on page7, and line 145-158 now reads, “The HH questionnaire includes socio–demographic characteristics (maternal age, marital status, maternal educational status, partner educational status, maternal occupation, partner occupation, place of residence, availability of radio and/or television in HH), reproductive health history (gravidity, parity, history of abortion, history of still birth, antenatal care (ANC), place of delivery and postnatal care (PNC)) and others related information (**S1 File**), and the data were collected through face–to–face interview.

Additionally, checklists were adapted from WHO assessment tools, national guideline and related studies [23,31–33], to collect on health facility data. Data on the availability of the ANC tracer items like: access to emergency transport, blood pressure apparatus, fetal stethoscope, adult weighing scale, examination bed, tape measure, hemoglobin tests, urine protein tests, syphilis test HIV tests kit, iron-folate tablets and tetanus toxoid vaccine were collected by interviewing health care workers at the facilities, and/or through direct observation using a predefined checklist (**S2 File**)”.

**Editor ’s comment:** Some details (e.g., exclusion of districts due to security issues) are mentioned but not fully explored in terms of their potential impact on the findings.

**Response to editor’s comment:** Thank you for your comment, we have already mentioned the potential impact on the limitation section on page 20, and line 375-378 now reads, Furthermore, some districts were not included in this study due to security reasons, and the proportion of effective coverage of ANC in the region might be lower than the coverage estimated in this study.

**2.1 Sampling Technique**

**Editor ’s comment:** The use of a multistage cluster sampling method is appropriate for a study of this scope and geography. However, your justification for selecting 30% of Tabias and 30 households per Tabia could be made more clear. While resource constraints are valid, citing additional evidence or methodological guidelines (beyond referencing the EDHS standards) would strengthen this decision.

**Response to editor’s comment:** Thank you for your valuable comment. We have added additional references as suggested.

The revised methods section (Sample size and sampling procedure sub-section) on page 6, and line 128-132 now reads, “Selecting 30% of Tabias (clusters) and 20–30 HHs per Tabia (cluster) is an effective approach for achieving statistical precision while maintaining resource efficiency in household surveys using a multi-stage cluster sampling method [30–32]”.

**Editor ’s comment:** The claim that 8 urban and 16 rural districts were selected "by chance" could use more elaboration, as this may raise concerns about the balance and representation of the sampling frame.

**Response to editor’s comment:** Thank you for your comment. We have lists of all 78 accessible districts in Tigray and then we randomly selected 24 districts using the lottery method to include in the study and finally 8 urban and 16 rural districts were selected.

**Editor ’s comment:** please ensure clarity on the randomization process used at each stage (districts, Tabias, households). A flowchart was added as per the editor’s suggestion, which helps, but detailed descriptions would enhance transparency.

**Response to editor’s comment:** Thank you for your comment, but we have already included as **fig 1** in the revised manuscript.

**2.2 Sample Size Determination**

**Editor ’s comment:** While the manuscript provides some rationale for the sample size (e.g., EDHS standards and resource constraints), a clearer power calculation or justification based on the study’s primary outcomes would strengthen the methodological rigor.

**Response to editor’s comment:** Thank you, we have added some justification in the revised manuscript.

**Editor ’s comment:** The selection of 250 ANC clients (15 per primary hospital and 5 per health center) appears arbitrary. The justification provided (based on client load) is insufficient and should be supported with references or additional details.

**Response to editor’s comment:** Thank you for your comment. Honestly, we did not use any references in the selection of 250 ANC clients (15 per primary hospital and 5 per health center). We planned to include a sample of 250 ANC clients based on an expert consensus because of cost effectiveness and then we selected an average of 5 clients from each health center and 15 clients from each primary hospital by systematic random sampling by considering the ANC client load from the previous three months to assess ANC quality process components.

**Editor ’s comment:** Please genuinly address why weighting was not applied to account for multi stage sampling, as noted by the reviewers. Even if you assume equal probabilities of selection, explicitly discussing this decision would preempt concerns about representativeness.

**Response to editor’s comment:** Thank you for your valuable feedback. We have addressed the issue of sample weighting in the revised limitations section, as suggested by the reviewers.

The revised methods discussion (strengths and limitations of the study sub-section) on page 20, and nd line 373-375 now reads, “Additionally, we did not consider sample weighting to account for the unequal probabilities of selection associated with the multi-stage cluster sampling method. This oversight may have affected the representativeness of our findings”.

**3.Discussion**

**Editor ’s comment:** The discussion is repetitive in places and could be restructured for clarity. For instance, comparisons with pre-war data and other conflict-affected regions should be consolidated into one cohesive argument rather than scattered across multiple paragraphs.

**Response to editor’s comment:** Thank you for your comment. As you may have noticed, we present multiple indicators related to antenatal care (ANC) service utilization, including ANC1, early initiation of ANC, and ANC4, among others. Consequently, we have tried to discuss each indicator in the context of pre-war data and findings from other conflict-affected areas, which is why you may see multiple information presented in various sections.

**Editor ’s comment:** While the authors highlight the uniqueness of the Tigray conflict, they could delve deeper into how this impacts ANC differently compared to other conflict settings. For example, the widespread looting and destruction of health facilities could be linked to specific quality gaps observed in the study.

**Response to editor’s comment:** Thank you for your comment**,** but we have already included in the consecutive sentence. These factors could be the possible reasons for the low coverage early ANC booking and ANC4+ in the region.

**4. Recommendations**

**Editor ’s comment:** The recommendations lack specificity in some areas. For instance, while promoting early ANC booking is essential, practical strategies to address barriers (e.g., transport issues, health facility readiness) could be more explicitly stated.

**Response to editor’s comment:** Thank you for your comment**.** We have tried to modify our recommendations as you suggested.

**Editor ’s comment:** Suggestions for training healthcare providers are too broad. Details about the specific gaps in provider competencies and the type of training required would make it actionable.

**Response to editor’s comment:** Thank you for your insightful comment. We have revised as you suggested.

**Editor ’s comment:** The call to improve facility readiness is valid, but given resource constraints in post conflict settings, prioritization of specific interventions could be emphasized.

**Response to editor’s comment:** Thank you for your insightful comment. We have revised as suggested.

**Editor ’s comment:** Recommendations should clearly align with the findings of the study. For example, the observed drastic drop off from ANC1 to ANC4+ coverage should be linked to targeted interventions to address this continuity gap.

**Response to editor’s comment:** Thank you for your comment. We have tried to link our findings with our recommendation as suggested.

**Reviewers' comments:**

**Reviewer #1**

**Reviewer’s comment:** The authors responded the following "In our sampling strategy, all individuals or households within the sampled Tabias were selected with equal probability using simple random technique." However, inside the document, I did not see a section that states simple random sampling was used. Also, the only way all the participants could have equal probability of being selected without using sample weighting is, if you conduct simple random sampling of the individuals, which is impossible, because it is not feasible to list all individuals from 24 districts. All districts have different number of tabyas and all tabyas have different number of household, and all households have different number of individuals, which is why sample weighting is very crucial here. So, I suggest, if sample weighting is not feasible at this time, the authors should at least mention this as a limitation.

**Response to reviewer’s comment**: Thank you for your insightful comment. We have incorporated the issue of sample weighting into the revised limitations section, as you suggested.

**Reviewer #3**

**Reviewer’s comment:** 1. Sampling Technique: The methodology regarding participant selection is not clearly described. The authors should provide a detailed explanation of the sampling process and illustrate the number of participants recruited from each district using a diagram.

**Response to reviewer’s comment**: Thank you for your comment, but we have already included the sampling procedure by diagram in **Fig 1.**

**Reviewer’s comment:** 2. Data Quality Assurance: The manuscript states that the questionnaire underwent a pre-test in a different location to assess response accuracy and estimate the required time. Please specify the location where the pre-test was conducted and describe any adjustments made based on the pre-test results.

**Response to reviewer’s comment**: Thank you, the comment is well accepted. We have mentioned the location which pre-test was conducted and the adjustments we made based on the pre-test results.

The revised methods discussion (data quality assurance sub-section) on page 8, and line 168-171 now reads, “Prior to commencing the actual survey, the tools were pre–tested in Adigrat town to evaluate response accuracy and estimate the required time. Based on the pre–test results, necessary adjustments like skipping pattern, coherence of the questions and options for multiple response were made correction before the actual data collection commenced”.

**Reviewer’s comment:** 3. The results are well-articulated and clearly presented.

**Response to reviewer’s comment**: Thank you very much for you recognition to our results.

**Reviewer’s comment:** 4. Given the reported low effective coverage of antenatal care (ANC) services in post-war Tigray, it is important to specify the baseline used for comparison. Clarifying this will help contextualize the findings.

**Response to reviewer’s comment**: Thank you for your comment, but we have already compared with pre–war in Tigray (38.7%) and national levels (12%–21.9%) [6,26].

**Reviewer’s comment:** 5. Strengths and Limitations: The section discussing the strengths and limitations of the study is not well developed. A thorough revision is needed to clearly highlight the key strengths and potential limitations.

**Response to reviewer’s comment**: Thank you for your comment. We have made a revision of the strengths and limitations section accordingly.

**Reviewer’s comment:**6. It would be beneficial for the authors to suggest directions for future research based on the study’s findings.

**Response to reviewer’s comment**: Thank you for your valuable comment. We have recommended future research in the revised manuscript.

**Reviewer’s comment:** 7. The conclusion does not fully reflect the study’s key findings. A thorough revision is needed to ensure alignment with the results and key discussions.

**Response to reviewer’s comment**: Thank you for your comment. We have revised as you suggested.

**Reviewer’s comment:** 8. References: DOIs should be included for all referenced articles where applicable

**Response to reviewer’s comment**: Thank you for your valuable comment. We have included DOIs as you suggested.
