# Supplementary material for: Effective coverage of antenatal care services in post war Tigray, Northern Ethiopia: An analysis of community and health facility–based surveys
Source: PLoS One. 2025 Oct 30;20(10):e0336121. doi: 10.1371/journal.pone.0336121 (PMC12574916; doi:10.1371/journal.pone.0336121)
Supplement: Attachment — Submitted filename: Responses_to_reviewers_auresp_3.docx [file pone.0336121.s009.docx]

**Responses to editor’s and reviewers’ comments**

We are writing to express our sincere gratitude to both the editor and the reviewers for your invaluable and insightful comments. Your feedback has been instrumental in significantly enhancing the quality of our manuscript to reach this stage.

We have carefully addressed all comments and incorporated the necessary revisions. Our manuscript has also been formatted according to the PLOS ONE style guidelines to ensure full compliance. Accordingly, we are pleased to resubmit a revised version of the manuscript and revised manuscript with tracked changes for your easy reference, along with this a detailed point-by-point response letter addressing each comment.

Thank you very much, looking forward to your favorable response

Below is our point-by-point response to the editor’s and reviewers’ comments for your kind consideration.

**Editor comments**:
**Editor ’s comment:** 1. # the first concern is the use of the term "post-war Tigray". While it is well justified in the contextual timeline of the study, caution must be taken given that the conflict dynamics were continuing throughout the period of this study. This means while open hostilities may have officially ended, insecurity persists in some zones. Thus, Using “post-war” may oversimplify ongoing humanitarian and systemic challenges including incomplete health system recovery. If the authors want to retain the term “post-war”, I suggest adding a clarification sentence in the Introduction or Methods section. Or they may acknowledge that the post-war label may not universally apply across all districts.

**Response to editor’s comment:**

Thank you for this important and thoughtful observation. We acknowledge that referring to the period as “post-war” may risk oversimplifying the complex and evolving security and humanitarian situation in Tigray. However, we have used the term “post-war” specifically in reference to the period following the signing of the Pretoria Peace Agreement in November 2022, which formally marked the cessation of hostilities.

To address this concern, we have added a clarifying sentence in the Introduction page 5 and lines number 107 -110), now reads, “…. While the term “post-war” is used to frame the study period following the formal cessation of hostilities, it is important to note that pockets of insecurity and systemic disruptions persist in some areas. Therefore, the term may not fully capture the diverse realities experienced across all zones in the region.”

**Editor ’s comment:** 2. # the second concern is in the measurement of effective coverage. While the omission of outcome-adjusted and adherence-adjusted coverage in the measurement of the effective coverage is clearly acknowledged, they are advised to acknowledge sensitivity of the multiplicative method (ANC4+ × readiness × intervention coverage × process quality) to small changes in any variable, which may lead to very low estimates (3.8% in this case). In addition giving each component the equal weight may not reflect their real-world impact (e.g., process quality may matter more than one missing tracer item). The use of Iron-folate supplementation as the sole intervention in the calculation of "intervention coverage" also need clarification as it may underestimate the broader scope of ANC content. In addition giving each component the equal weight may not reflect their real-world impact (e.g., process quality may matter more than one missing tracer item). The use of Iron–folate supplementation as the sole intervention in the calculation of "intervention coverage" also may underestimate the broader scope of ANC content

**Response to editor’s comment:**

We sincerely appreciate this valuable feedback. We agree that the multiplicative method for estimating effective coverage is inherently sensitive to small variations in any of its components (ANC4+, facility readiness, intervention coverage, and process quality), which can result in a lower overall estimate. This aligns with the intuitive calculations of effective coverage as recommended by the Measurement of Effective Coverage Think Tank Group (1). Furthermore, World Health Organization (WHO) Service Availability and Readiness Assessment (SARA) uses equal weighting when it calculates facility reediness and quality process (2). In fact, studies have shown that equal weighting and expert-based weighting approaches yield similar index scores; however, because expert ratings can vary across expert groups, the equal-weighting method is generally preferred due to its simplicity, replicability, and endorsement by WHO (3).

While we acknowledge that assigning equal weights to all components may not fully reflect their relative real-world impact, we adopted this approach in the absence of standardized or validated weighting schemes in the literature. Equal weighting also facilitates comparability with other studies and ensures a pragmatic and transparent methodology.

Regarding the use of iron–folate supplementation for intervention coverage, we selected it as the sole proxy indicator for ANC intervention coverage based on three considerations: (1) the consistent availability of iron–folate supplementation data across all gestational ages in the dataset; (2) its universal recommendation by the World Health Organization (WHO) as a critical component of ANC; and (3) its widespread implementation and documentation, which facilitates comparability across settings.

We recognize, however, that relying solely on iron–folate supplementation may underestimate the full spectrum of ANC content, as it excludes other essential interventions. To our knowledge, there is no strict requirement to use multiple interventions for calculating effective coverage; alternatively, it can be estimated without selecting a specific intervention by multiplying the average quality of service by crude (contact) coverage (4).

**To support our approach, we refer to the following key sources:**

1. Marsh AD, Muzigaba M, Diaz T, Requejo J, Jackson D, Chou D, et al. (2020). Effective coverage measurement in maternal, newborn, child, and adolescent health and nutrition: progress, future prospects, and implications for quality health systems. Lancet Global Health, 8(5).
2. WHO. Service Availability and Readiness Assessment (SARA): An annual monitoring system for service delivery implementation Guide. World Heal Organ ;2015.
3. World Health Organization. (2015). Tracking universal health coverage: First global monitoring report. Geneva: World Health Organization and the World Bank.
4. Yakob B, Gage A, Nigatu TG, Hurlburt S, Hagos S, Dinsa G, et al. Low effective coverage of family planning and antenatal care services in Ethiopia. Int J Qual Heal Care. 2019;31(10):725–32.

**Reviewers' comments:
Reviewer #4:**

**Reviewer’s comment:** 1. A justification statement that appear at line 98-100, which indicates the inconsistency of the previous study findings, is not an appropriate justification for a primary study. Such kinds of justifications will be addressed by conducting a review (like Systematic review and/or Meta analysis) of the previous inconsistent studies not by primary study like the current one.

**Response to reviewer’s comment**:

Thank you for your valuable comment. We agree that when inconsistencies in previous study findings are observed, a systematic review or meta-analysis is generally the more appropriate approach to address such discrepancies. In our manuscript, we referred to the inconsistency as part of describing the overall knowledge gap, noting that the available evidence is both scarce and varied. However, our primary aim was not to assess the consistency of previous findings. Instead, the main objective of our study was to evaluate the effective coverage of ANC services in the post-war context of the Tigray Region, where no sufficient primary data currently exists.

**Reviewer’s comment:** 2. The introduction section better address the significance of the study.
**Response to reviewer’s comment**: Thank you for your insightful comment. We have added as suggested.

The revised introduction section on page 5 and line 112-115 now reads, “The findings will provide crucial evidence to guide policymakers, program managers, and healthcare providers in prioritizing interventions, allocating resources, and rebuilding resilient health systems to improve ANC effective coverage in post-conflict settings.”

**Reviewer’s comment:** 3. You used very old data, almost 20 years ago, to indicate the population size of Tigray (line 116). I would rather recommend to use the estimated one (if available) since we do not have a recent census.
**Response to reviewer’s comment**: Thank you for your comment. We have rephrased the sentence accordingly. We have used the projections from the 2007 census because there is not census after 2007 in Ethiopia.

The revised methods section and Study design, setting and period sub section on page 6 and line 121-123 now reads, “According to the 2007 census, the total population of Tigray in 2024 is estimated at approximately 7 million, with around 80% residing in rural areas.”

**Reviewer’s comment:** 4. You better use an appropriate English term for "Tabias" at line 121 and other lines. It can be "Sites" or any other.
**Response to reviewer’s comment**: Thank you for your comment. We have revised as you suggested “Tabias (the smallest administrative units in Ethiopia).”

**Reviewer’s comment:** 5. The study population, specially facility based study population, are vague as well as contradictory with the consequent descriptions. It is stated as if the facilities are the study population (line 121-123) but it is not like that as stated in the next sections and according to the general essence of the study.
**Response to reviewer’s comment**: Thank you observation. We have revised as the following in the revised manuscript “the study population for the health facility–based study included ANC clients from all primary hospitals and health centers providing services to these women.”

**Reviewer’s comment:** 6. Your sampling procedure is unclear.
Eg. line 128: "..... 2340 women (78 Tabias x 30 Households per tabia).......". The computation in the bracket will give number of households in the the 78 Tabias not the number of women. Or did you assume 1 women in a household?
**Response to reviewer’s comment**: Thank you for your insightful comment. We have changed the households to women in the revised methods section and Sample size and sampling procedure sub section on page 6 and line 131-133 now reads, “A total sample size of 2340 women (78 Tabias × 30 women per Tabia) who give birth within 12 months prior to data collection were included in this study.”

**Reviewer’s comment:** 7. You better specify the type of observation you used at line 160-161. Is it overt or covert(concealed)? If it is overt, how did you manage the bias?

**Response to reviewer’s comment**:

Thank you for your insightful comment. The observation conducted in our study was **overt,** meaning that participants were aware that they were being observed. To minimize observation bias, we ensured that data collectors were trained to be as unobtrusive and neutral as possible during the observation process. Additionally, the observers maintained consistent behavior across all sessions to reduce the biases. We have already stated this in the in methods section and data collection tools and techniques sub section in the revised manuscript.
